# An Improved Opposition-Based Learning Particle Swarm Optimization for the Detection of SNP-SNP Interactions

**DOI:** 10.1155/2015/524821

**Published:** 2015-07-05

**Authors:** Junliang Shang, Yan Sun, Shengjun Li, Jin-Xing Liu, Chun-Hou Zheng, Junying Zhang

**Affiliations:** ^1^School of Information Science and Engineering, Qufu Normal University, Rizhao 276826, China; ^2^Bio-Computing Research Center, Shenzhen Graduate School, Harbin Institute of Technology, Shenzhen 518055, China; ^3^College of Electrical Engineering and Automation, Anhui University, Hefei, Anhui 230039, China; ^4^School of Computer Science and Technology, Xidian University, Xi'an 710071, China

## Abstract

SNP-SNP interactions have been receiving increasing attention in understanding the mechanism underlying susceptibility to complex diseases. Though many works have been done for the detection of SNP-SNP interactions, the algorithmic development is still ongoing. In this study, an improved opposition-based learning particle swarm optimization (IOBLPSO) is proposed for the detection of SNP-SNP interactions. Highlights of IOBLPSO are the introduction of three strategies, namely, opposition-based learning, dynamic inertia weight, and a postprocedure. Opposition-based learning not only enhances the global explorative ability, but also avoids premature convergence. Dynamic inertia weight allows particles to cover a wider search space when the considered SNP is likely to be a random one and converges on promising regions of the search space while capturing a highly suspected SNP. The postprocedure is used to carry out a deep search in highly suspected SNP sets. Experiments of IOBLPSO are performed on both simulation data sets and a real data set of age-related macular degeneration, results of which demonstrate that IOBLPSO is promising in detecting SNP-SNP interactions. IOBLPSO might be an alternative to existing methods for detecting SNP-SNP interactions.

## 1. Introduction

There is an increasing interest in understanding the underlying genetic architecture of complex diseases, such as cancer, heart disease, diabetes, Crohn's disease, and many others, which represent the major part of current clinical diseases [1, 2]. Research of complex diseases is one of the hottest fields of bioinformatics and genome-wide association studies (GWAS) become routine strategies. With the methods of GWAS, hundreds of thousands of single nucleotide polymorphisms (SNPs) speculated to associate with complex diseases have been identified. Nevertheless, these SNPs have limited effects on predicting the phenotype, and a large fraction of genetic contributions to complex diseases remain unclear. Recent advances make it clear that besides rare SNPs not genotyped in GWAS, the “missing heritability” can be partly explained by nonlinear interactive effects of multiple SNPs, namely, SNP-SNP interactions [3]. Detection of SNP-SNP interactions is therefore a compelling next step in GWAS.

In general, the detection of SNP-SNP interactions is a great challenge [4]. The first challenge is the intensive computational burden imposed by the enormous search space, which prohibits real applications of most existing methods, especially those exhaustive ones. For instance, search space of a 100,000-SNP data set with considered maximum order of 3 is an astronomical number ∑_*k*=1_^3^ *C*_100,000_^*k*^. The second challenge is the complexity of genetic architecture of a complex disease. Limited or even no prior knowledge available for a complex disease, such as the order and the effect magnitude of a SNP-SNP interaction, makes it difficult for the development of heuristic methods. The third evaluation measures that determine how well a SNP combination contributes to the phenotype are limited. Evaluation measures should be efficient in computational cost and insensitive to both SNP combination order and dependency type. Though several evaluation measures have been widely used in the detection of SNP-SNP interactions, developing new evaluation measures that can effectively and efficiently capture SNP-SNP interactions is still a direction.

Though methodological and computational perplexities of the detection of SNP-SNP interactions have been well recognized, the algorithmic development is still ongoing. Exhaustive algorithms, for example, MDR [5], appear promising for small scale data sets. However, for large scale data sets, especially those for GWAS, the detection of SNP-SNP interactions becomes a* needles-in-a-haystack* problem and exhaustive algorithms lose their ability [6, 7]. Heuristic algorithms are popular since they can retain as many informative SNPs as possible while largely reducing computational complexity. For example, Jiang et al. formulated the detection of SNP-SNP interactions from the viewpoint of binary classification and designed* epi*Forest on the basis of the gini importance given by the random forest to select a small set of candidate SNPs [8]. Zhang and Liu proposed a Bayesian partition approach BEAM to find groups of genotypes with large posterior probability [9]. Tang et al. introduced the concept of epistatic module and designed a Gibbs sampling approach *epi*MODE to detect such modules [10]. Wan et al. developed a SNP-SNP interaction detection method SNPRuler based on both predictive rule inference and two-stage design [11]. They also presented another method BOOST, which involves only Boolean values and allows the use of fast logic operations to obtain contingency tables [12]. Besides machine learning methods, entropy based methods are also applied to this field. Chanda et al. developed an interaction index based on entropy theory to prioritize interacting SNPs [13]. They also applied two entropy theoretic measures to three SNP-SNP interaction detection methods: AMBIENCE [14] with a phenotype associated information measure; KWII [15] with the coinformation measure detecting SNP-SNP interactions associated with the binary phenotype; and CHORUS [16] combining these two measures together to identify associations with quantitative traits.

Recently, many swarm intelligence based algorithms have been proposed for the detection of SNP-SNP interactions [17–31]. Among them, particle swarm optimization (PSO) appears promising and some related works have been reported [22–30]. Yang et al. [22] used the binary PSO with odds ratio as the fitness function (OR-BPSO) to evaluate the risk of breast cancer. Based on the OR-BPSO, Chang et al. [23] proposed the odds ratio-based discrete binary PSO (OR-DBPSO) for the detection of SNP-SNP interactions with the quantitative phenotype. Chuang et al. [24] proposed a chaotic PSO (CPSO) that identifies the best SNP combination for breast cancer association studies. For enhancing the reliability of the PSO in the identification of the best SNP-SNP interaction associated with breast cancer, they also developed an improved PSO (IPSO) [25] and proved that the IPSO is highly reliable than the OR-BPSO. More recently, they used the gauss chaotic map PSO (Gauss-PSO) to detect the best association with breast cancer [26]. Experimental results revealed that the Gauss-PSO was able to identify higher difference values between cases and controls than both the PSO and the CPSO. Yang et al. [27] developed a double-bottom chaotic map PSO (DBM-PSO) that overcomes the respective disadvantages of the PSO and the CPSO. Then, DBM-PSO is successfully applied to determine gene-gene interactions based on* chi*-square test [28]. Hwang et al. [29] proposed a complementary-logic PSO (CLPSO) to increase the efficiency of significant model identification in case-control study. Wu et al. [30] applied PSO to analyze the SNP-SNP interactions associated with hypertension. However, these methods, almost all of which are developed by a group except the one proposed by Wu et al. [30], only focus on finding the best genotype-genotype of a SNP-SNP interaction among possible genotypes of SNP combinations, but not the SNP-SNP interactions among possible SNP combinations. Obviously, the limited sample size of SNP data affects their computational accuracies of fitness functions and hence hinders their further applications. Furthermore, these methods are experimented on very small scale data sets (<30 SNPs) of certain complex diseases, performance of which on various kinds of large scale data sets are still unclear.

In this study, we proposed an improved opposition-based learning particle swarm optimization (IOBLPSO) with mutual information as its fitness function to detect SNP-SNP interactions. IOBLPSO is the first PSO based method to find SNP-SNP interactions among possible SNP combinations. Highlights of IOBLPSO are the introduction of three strategies, that is, opposition-based learning (OBL), dynamic inertia weight, and a postprocedure. Among them, OBL is the core, which is presented in the stage of updating particle experiences and common knowledge of swarm, not only for enhancing the global explorative ability, but also for avoiding premature convergence. Dynamic inertia weight is computed before the stage of updating particle velocities to allow particles to cover a wider search space when the considered SNP is likely to be a random SNP and to converge on promising regions of the search space while capturing a highly suspected SNP. The postprocedure is used as the final stage for carrying out a deep search in highly suspected SNP sets. Experiments of IOBLPSO are performed on lots of simulation data sets under the evaluation measures of both detection power and computational complexity. Results demonstrate that IOBLPSO is promising for the detection of all simulation models of SNP-SNP interactions. IOBLPSO is also applied on data set of age-related macular degeneration (AMD). Results show the strength of IOBLPSO on real applications and capture important features of genetic architecture of AMD that have not been described previously, which provide new clues for biologists on the exploration of AMD associated SNPs. IOBLPSO might be an alternative to existing methods for the detection of SNP-SNP interactions.

## 2. Methods

### 2.1. Particle Swarm Optimization (PSO)

The PSO, proposed by Kennedy and Eberhart [32], is a member of the family of swarm intelligence algorithms, which mimic the collective behaviors of organisms based on information sharing, like ants and birds, which can jointly perform many complex tasks though each individual is very limited in its capability. The PSO is a stylized representation of the movement of birds (viewed as particles) in a flock, where each particle uses its own experience and the common knowledge gained by the entire swarm to find an optimal position [29].

In PSO, the position of a particle represents a possible solution. In each generation, the position of each particle is adjusted according to its updated velocity and is estimated by a fitness function for providing a good search direction. Whether the velocity of each particle is updated depends on three variables: its previous velocity, its individual experience, and the common knowledge of the swarm. Specifically, the individual experience of each particle is updated while fitness value of its current position is higher than that of its previous experience; the common knowledge of the swarm is updated by the one of individual experiences of all particles with the highest fitness value while such value is higher than that of their previous common knowledge. This feedback strategy leads the swarm to gradually converge to an optimal solution [25–29].

Owing to its high capability and good generality in solving complex problems, the PSO has become a widely adopted swarm intelligence algorithm. However, it still has several defects, for example, premature convergence, stagnation phenomenon, and slow convergence speed in the later evolution period, which imply that the PSO should be further improved, especially for a specific complex problem, for example, the detection of SNP-SNP interactions. In general, the PSO consists of 4 stages: (1) initializing particles, (2) evaluating particles using fitness function, (3) updating particle velocities and positions, and (4) updating particle experiences and common knowledge of swarm. These stages are detailed and described in the following section.

### 2.2. IOBLPSO: An Improved Opposition-Based Learning Particle Swarm Optimization for the Detection of SNP-SNP Interactions

**Figure 1:**
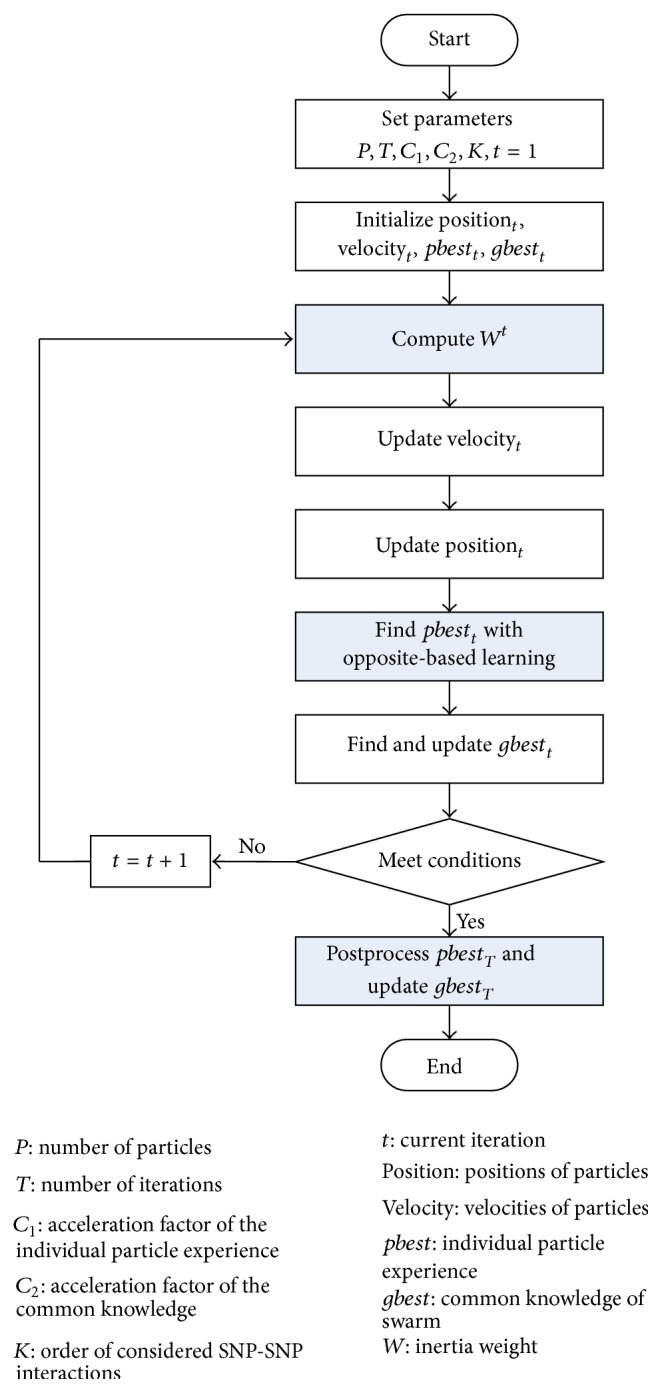
The flowchart of IOBLPSO. Three components with grey background are highlights of IOBLPSO.

The flowchart of IOBLPSO is shown in Figure 1, where its highlights are with grey background. Below we describe IOBLPSO in detail from 6 stages.

*(1) Mapping SNPs and Initializing Particles*. At present, the popular way of mapping SNPs is to collect them as a matrix, where a row represents genotypes of an individual and a column represents a SNP. Genotypes of a SNP are coded as {0,1, 2}, corresponding to homozygous common genotype (e.g., *AA*, *BB*), heterozygous genotype (e.g., *Aa*, *aA*, *Bb*, *bB*), and homozygous minor genotype (e.g., *aa*, *bb*). The label of an individual is a binary phenotype being either 0 (control) or 1 (case).

Based on above numerical mapping, the position of the *p*_*th*_ particle at iteration *t* can be represented as Position_*t*_(*p*) = (SNP_*p*1_^*t*^,…, SNP_*pk*_^*t*^,…, SNP_*pK*_^*t*^), where *p* ∈ {1,2,…, *P*}, *k* ∈ {1,2,…, *K*}, *t* ∈ {1,2,…, *T*}, *P* is the number of particles, *K* is the considered order of SNP-SNP interactions, *T* is the number of iterations, SNP_*pk*_^*t*^ is the index of the selected *k*_*th*_ SNP of the *p*_*th*_ particle at iteration *t*, SNP_*pk*_^*t*^ ∈ {1,2,…, *M*}, and *M* is the number of SNPs in the data set. The velocity of the *p*_*th*_ particle at iteration *t* is represented as Velocity_*t*_(*p*) = (*v*_*p*1_^*t*^,…, *v*_*pk*_^*t*^,…, *v*_*pK*_^*t*^), where *v*_*pk*_^*t*^ is the velocity of SNP  SNP_*pk*_^*t*^ and *v*_*pk*_^*t*^ ∈ [1 − *M*, *M* − 1]. Similarly, the individual experience of the *p*_*th*_ particle, that is, the position of the *p*_*th*_ particle with the highest fitness value until iteration *t*, can be denoted as *pbest*_*t*_(*p*) = (*p*SNP_*p*1_^*t*^,…, *p*SNP_*pk*_^*t*^,…, *p*SNP_*pK*_^*t*^), and the common knowledge of swarm, that is, the best position of all particles with the highest fitness value until iteration *t*, is denoted as *gbest*_*t*_ = (*g*SNP_1_^*t*^,…, *g*SNP_*k*_^*t*^,…, *g*SNP_*K*_^*t*^).

Before the first iteration, Position_1_(*p*), Velocity_1_(*p*), *pbest*_1_(*p*), and *gbest*_1_ are randomly initialized in their respective domains.

*(2) Updating Dynamic Inertia Weight*. Inertia weight is used to control the impact of the previous velocity of a particle on its current velocity. A large inertia weight facilitates the global exploration and thus enables the method to execute a search over various regions, while a small inertia weight facilitates the local exploitation, which searches a promising region [27]. In order to effectively balance the global exploration and the local exploitation, a dynamic inertia weight is introduced to IOBLPSO, which can be defined as

(1) $$\begin{array}{c} W_{pk}^{t}=\frac{max\left({count}_{t}\right)-{count}_{t}\left[p{SNP}_{pk}^{t}\right]}{max\left({count}_{t}\right)-min\left({count}_{t}\right)}, \end{array}$$

where count_*t*_ = (ct_1_^*t*^,…, ct_*m*_^*t*^,…, ct_*M*_^*t*^) and ct_*m*_^*t*^ is a counter that counts the number of SNP *m* presented in *pbest* from iteration 1 to iteration *t*. This strategy allows particles to cover a wider search space while the considered SNP is likely to be a random SNP and to converge on promising regions of the search space while capturing a highly suspected SNP.

*(3) Evaluating Particles Using Fitness Function*. Fitness function of the IOBLPSO plays an important role on deciding which SNP combination is the SNP-SNP interaction and measuring how much the effect of a captured SNP-SNP interaction to the phenotype is. In the IOBLPSO, mutual information is applied as its fitness function, since it is well developed and can measure multivariate dependence without complex modeling. Mutual information has been widely used as a promising measure for feature selection and here is defined as

(2) $$\begin{array}{c} MI\left(\;X;Y\;\right)=H\left(\;X\;\right)+H\left(\;Y\;\right)-H\left(\;X,Y\;\right), \end{array}$$

where *H*(*X*) is the entropy of *X*; *X*, representing a SNP combination, is the general expression of Position_*t*_(*p*), *pbest*_*t*_(*p*), and *gbest*_*t*_; *H*(*Y*) is the entropy of the phenotype *Y*; *H*(*X*, *Y*) is the joint entropy of both *X* and *Y*. It is clear that higher mutual information value, namely, fitness value, indicates stronger association between the phenotype and the SNP combination.

*(4) Updating Particle Velocities and Positions*. IOBLPSO executes a search for SNP-SNP interactions by continuously updating particle velocities and particle positions in all iterations. The velocity of SNP_*pk*_^*t*^ is updated using the following equations:

(3) v~pkt+1=Wpkt·vpkt+C1·r1·pSNPpkt−SNPpkt+C2·r2·gSNPkt−SNPpkt,vpkt+1=v~pkt+1v~pkt+1∈1−M,M−1rand1−M,M−1v~pkt+1∉1−M,M−1,

where *C*_1_ and *C*_2_, controlling how far a particle moves in a single iteration, are acceleration factors and *r*_1_ and *r*_2_ are random values in (0,1). To obtain a valid velocity, a random value is sampled in [1 − *M*, *M* − 1] while ${\tilde{v}}_{pk}^{t+1}$ exceeds its domain. Based on *v*_*pk*_^*t*+1^, the position of SNP_*pk*_^*t*^ can be updated by the following two equations:

(4) SNP¯pkt+1=SNPpkt+vpkt+1,SNPpkt+1=intSNP¯pkt+1SNP¯pkt+1∈1,Mintrand1,MSNP¯pkt+1∉1,M.

Because of SNP_*pk*_^*t*+1^ being a SNP index, an integer between 1 and *M* is randomly sampled if ${\bar{SNP}}_{pk}^{t+1}$ exceeds its domain. Such random sampling strategies on updating of both *v*_*pk*_^*t*+1^ and SNP_*pk*_^*t*+1^ help to increase the diversity of the search, the more possibility of jumping out local optima and getting into global optima.

*(5) Updating Particle Experiences and Common Knowledge of Swarm*. Another strategy introduced to IOBLPSO is the OBL. The basic principle of OBL is the consideration of a solution and its corresponding opposite solution simultaneously to approximate the global optima [33]. In the IOBLPSO, if the solution is Position_*t*_(*p*), its corresponding opposite solution can be defined as

(5) $$\begin{array}{c} {Position}_{t}^{\prime }\left(\;p\;\right)=1+M-{Position}_{t}\left(\;p\;\right). \end{array}$$

By comparing fitness values of Position_*t*_(*p*), Position_*t*_′(*p*), and *pbest*_*t*_(*p*), the individual experience of the *p*_*th*_ particle at iteration *t* + 1, that is, *pbest*_*t*+1_(*p*), is updated to the one among them with highest fitness value, which can be written as

(6) pbestt+1p =PositiontpMIPositiontp;Y=ValPositiont′pMIPositiont′p;Y=ValpbesttpMIpbesttp;Y=Val,

where Val = max⁡(*MI*(Position_*t*_(*p*); *Y*), *MI*(Position_*t*_′(*p*); *Y*), and *MI*(*pbest*_*t*_(*p*); *Y*)). From this equation, it can be seen that the employed OBL strategy facilitates IOBLPSO not only expanding the search space and enhancing the global explorative ability, but also accelerating the convergence and avoiding premature convergence.

Similarly, whether the common knowledge of the swarm at iteration *t* + 1, for example, *gbest*_*t*+1_, is updated or maintained as *gbest*_*t*_ depends on fitness values of individual experiences of all particles at iteration *t* + 1 and can be defined as

(7) gbestt+1=pbestt+1pMIpbestt+1p;Y>MIgbestt;YgbesttMIpbestt+1p;Y≤MIgbestt;Y.

*(6) Deep Searching with a Postprocedure*. A postprocedure is provided when completing the iteration process to carry out a deep search of SNP-SNP interactions in a highly suspected SNP sets. First, all SNPs are descending sorted according to their counters in count_*T*_, and the specified number of top SNPs (By default, 10) are selected into the highly suspected SNP sets. Second, IOBLPSO conducts an exhaustive search within the highly suspected SNP sets to determine whether fitness value of one or more SNP combinations is higher than that of *gbest*_*T*_. If indeed detected, *gbest*_*T*_ is updated by the best one among them. *gbest*_*T*_ is therefore the final result of IOBLPSO.

## 3. Results and Discussion

### 3.1. Simulation Data

**Figure 2:**
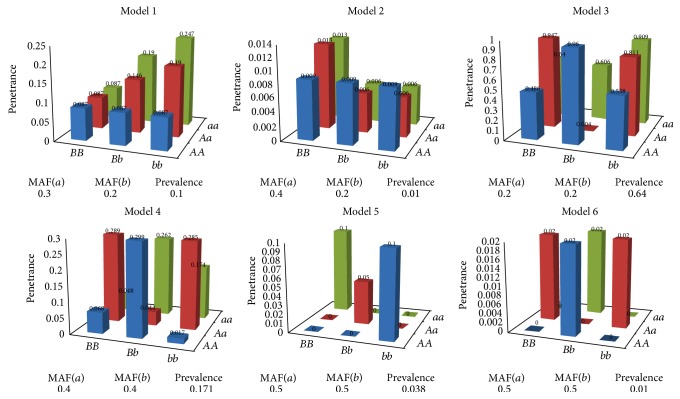
The simulation models of SNP-SNP interactions. In the figure, penetrance is the probability of the occurrence of a disease given a particular genotype; prevalence is the proportion of individuals that occur a disease; MAF(*a*) and MAF(*b*) are, respectively, minor allele frequencies of *a* and *b*.

Six commonly used models of SNP-SNP interactions with their orders being equal to 2 (i.e., *K* = 2) are exemplified for the study [7, 9, 10, 34, 35]. Model 1 and Model 2 are models displaying both marginal effects and interactive effects, and others show no marginal effects but interactive effects. Specifically, the penetrance in Model 1 increases only when both SNPs have at least one minor allele [9, 10]; Model 2 assumes that the minor allele in one SNP has the marginal effect; however the effect is inversed while minor alleles in both SNPs are present [9]; Model 3 and Model 4 are directly cited from the reference [35]; Model 5 is a ZZ model [34]; and Model 6 is an XOR model [35]. Model 3~Model 6 are exemplified here since they provide a high degree of complexity to challenge ability of a method in detecting SNP-SNP interactions [7]. For each model, 50 data sets are generated by the simulator EpiSIM [36], each containing 2000 cases and 2000 controls genotyped with 100 SNPs. For each data set, random SNPs are set independently with MAFs chosen from [0.05,0.5] uniformly and detailed parameters of ground-truth SNPs are recorded in Figure 2, where ground-truth SNPs refer to the causative SNPs that truly associated with the phenotype, in other words, the SNPs in models added into the simulation data sets.

### 3.2. Evaluation Measure

Detection power is one of the generally used evaluation measures in the field of the detection of SNP-SNP interactions, and various forms of detection power have been proposed depending on what is desired to measure [4, 7, 9–11, 21, 31, 37]. In this study, two types of detection power are introduced, namely, Power 1 and Power 2.

Power 1 [4, 7, 9–11, 21, 31] is defined as the proportion of data sets in which all ground-truth SNPs are detected with no false positives, which can be written as

(8) $$\begin{array}{c} \text{Power 1}=\frac{1}{N}\overset{N}{\underset{i=1}{\sum }}x_{i}, \end{array}$$

where *N* is the number of data sets with the same parameter settings (here, *N* = 50), and *x*_*i*_ ∈ {0,1} is the detection tag; that is, if 2 ground-truth SNPs in data set *i* are detected with no false positives, *x*_*i*_ = 1; otherwise, *x*_*i*_ = 0. Though Power 1 seems not practical since false positives are inevitable for any statistical tests and fewer false positives result in larger false negatives, we still introduce it because it is advantageous in practical applications and might be of interest to biologists due to false positives implying wasted experimental effort to validate the results.

Sometimes, allowing some small Type-I error rate is more reasonable; thus Power 2 [4, 7, 21] is introduced here, which is defined as an average proportion of ground-truth SNPs in the top 2 detected SNPs, and can be written as

(9) $$\begin{array}{c} \text{Power 2}=\frac{1}{2\cdot N}\overset{N}{\underset{i=1}{\sum }}y_{i}, \end{array}$$

where *y*_*i*_ is the number of ground-truth SNPs in the top 2 SNPs identified in data set *i*.

Computational complexity is also considered. We measure running time in the same computational environment to assess realistic applicability of compared methods.

### 3.3. Performance of IOBLPSO on Simulation Data

To demonstrate the validity of IOBLPSO, its detection power is evaluated by comparison with several typical SNP-SNP interaction detection methods, that is, BOOST [12], AntEpiSeeker [20], SNPRuler [11], and TEAM [38]. These machine learning methods are recently proposed, claimed to facilitate large scale data sets, and their packages are online freely available [7]. Besides these methods, two modified PSO methods for SNP-SNP interaction detection, namely, DBM-PSO [27] focusing on finding the best genotype-genotype of a SNP-SNP interaction among possible genotypes of SNP combinations and the PSO focusing on finding SNP-SNP interactions among possible SNP combinations, are also compared.

In the study, parameters of each method are generally set as default. Only a few are changed according to suggestions in order to balance result accuracy and computational cost. For BOOST, interaction threshold is set to 10, that is, results of BOOST are the SNP-SNP interactions whose likelihood ratio test statistic values >10 with 4 degrees of freedom. For AntEpiSeeker, the numbers of ants and iterations is set to 500 and 10, respectively. For TEAM, permutation number is set to 100. For a fair comparison, parameter settings of PSO based methods are the same. Specifically, the number of particles *P* and the number of iterations *T* are respective set to 100 and 100; both acceleration factors *C*_1_ and *C*_2_ are set to 2 [39]; the inertia weight *W* is set to 0.65. It is believed that performance of IOBLPSO mainly depends on parameters (*P*, *T*). Hence we further examine the influence of these parameters on detection power with (25,100), (50,100), (100,25), (100,50), and (100,100).

**Figure 3:**
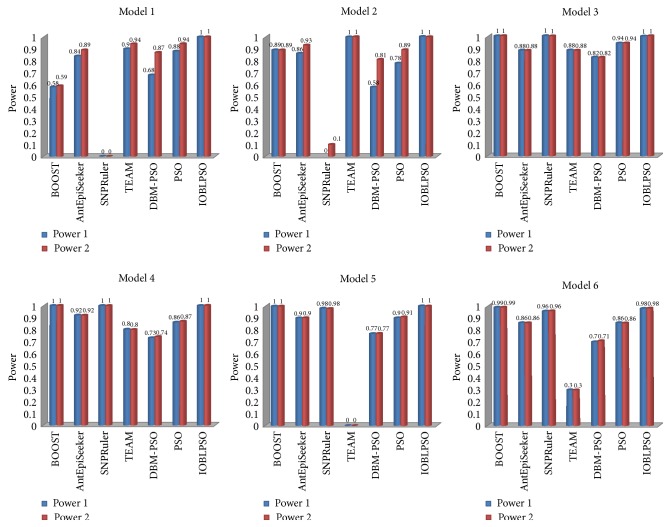
Detection power of compared methods on simulation data sets.

**Figure 4:**
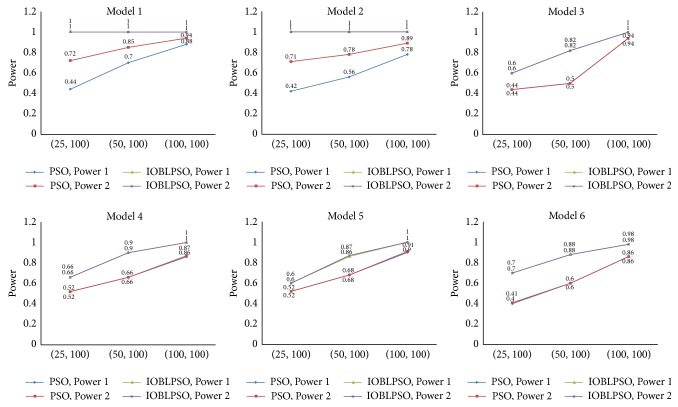
Detection power of IOBLPSO and the PSO with different numbers of particles. The numbers of particles are set to 25, 50, and 100, while the number of iterations is equal to 100.

**Figure 5:**
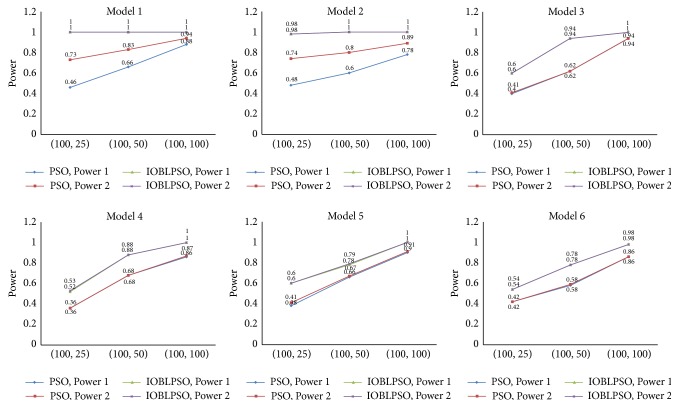
Detection power of IOBLPSO and the PSO with different numbers of iterations. The numbers of iterations are set to 25, 50, and 100, while the number of particles is equal to 100.

Detection power of compared methods on simulation data sets is reported in Figure 3. Detection power of IOBLPSO and the PSO with different numbers of particles is shown in Figure 4, and that with different numbers of iterations is shown in Figure 5. The average running time of the methods on simulation data sets is recorded in Table 1. From Figures 3, 4, and 5 and Table 1, we have the following observations.

It is seen that IOBLPSO outperforms compared methods on all cases regardless of models, the numbers of particles, and iterations. Specifically, no matter, according to Power 1 or Power 2, detection power of IOBLPSO on all models and (*P*, *T*) settings is comparable and sometimes superior to that of compared methods, which might be the result of introducing three effective strategies into IOBLPSO: OBL expanding the search space and enhancing the global explorative ability, dynamic inertia weight guiding the particles to more promising regions, and postprocedure carrying out a deep search in highly suspected SNP sets; with the numbers of particles or iterations grow, detection power of both IOBLPSO and the PSO increase quickly, especially IOBLPSO; IOBLPSO identifies almost all ground-truth SNPs on all models with the parameter setting (100,100), even with (25,100) or (100,25); IOBLPSO has perfect detection power on Model 1 and Model 2; that is to say, compared with other PSO based methods, IOBLPSO needs less particles and/or iterations to obtain higher detection power, implying that IOBLPSO can handle large scale data sets for GWAS and its scalability is better than others; for Model 1 and Model 2, Power 1 and Power 2 of IOBLPSO reach a prefect level, Power 1 and Power 2 of other methods have different values since these two models display not only interaction effects but also marginal effects, leading to compared methods sometimes only identifying several ground-truth SNPs, but not SNP-SNP interactions; similarly, for each method on Model 3~Model 6, Power 1 and Power 2 of each compared method are almost always equal because single ground-truth SNPs show no main effects; in terms of computational complexity, though IOBLPSO is not the fast one among all compared methods, it can finish the work at affordable time costs; more importantly, its time costs can be estimated and controlled by setting the numbers of particles and iterations freely under the premise of ensuring sufficient accuracy.

### 3.4. Application to Real AMD Data

In the study, potential of IOBLPSO can also be verified by analyzing a real AMD data set [40], which contains 103.611 SNPs genotyped with 96 cases and 50 controls. AMD, which refers to pathological changes in the central area of the retina, is the most important cause of irreversible visual loss in elderly populations and is considered as a complex disease whereby multiple SNP-SNP interactions interact with environmental factors to the disease [4, 10]. We run IOBLPSO on AMD data set 20 times with different combinations of the number of particles *P*  (10000,20000) and the number of iterations *T*  (500,1000), each running 5 times. The order of SNP-SNP interactions *K* is set to 2 since the small sample size of 146 individuals is insufficient for secure detection of any higher order SNP-SNP interactions. Both acceleration factors *C*_1_ and *C*_2_ are set to 2. Detected SNP-SNP interactions associated with AMD are listed in Table 2, where their mutual information values of individual SNPs and SNP-SNP interactions are recorded.

It has been widely accepted that rs380390 and rs1329428 are believed to be significantly associated with AMD [10]. These two SNPs are in an intron of the* CFH* gene in chromosome 1. There are biologically plausible mechanisms for the involvement of* CFH* in AMD and at least 100 mutations in* CFH* have been proven to increase the risk of AMD and other disorders.* CFH* is a regulator that activates the alternative pathway of the complement cascade, the mutations in which can lead to an imbalance in normal homeostasis of the complement system. This phenomenon is thought to account for substantial tissue damage in AMD [41]. In the IOBLPSO, these two SNPs are detected as members of SNP-SNP interactions, especially the rs380390. Almost all SNP-SNP interactions include rs380390, since it has the strongest main effect, leading to its combinations with other SNPs displaying strong interaction effects. This phenomenon indicates that IOBLPSO is sensitive to those SNPs displaying strong main effects.

The SNP-SNP interaction (rs380390, rs1374431), also reported by [42, 43], has the strongest interaction effect. Rs1374431 is located in a noncoding region between genes* LOC644301* and* KIAA1715*.* KIAA1715* is usually found in adult brain regions. Although no evidences were reported with this gene related to AMD, it may be a plausible candidate gene associated with AMD [42, 43]. Another SNP-SNP interaction (rs380390, rs2402053) has the second highest mutual information value. The SNP rs2402053 is in the intergenic region between genes* TFEC* and* TES* in chromosome 7q31 [44]. It is worth noting that mutations in some genes on 7q31-7q32 are revealed in patients with retinal disorders [45]. Therefore, rs2402053 may be a new genetic factor contributing to the underlying mechanism of AMD [46–50].

It is interesting that the SNP-SNP interaction (rs380390, rs1363688) was successfully detected 8 times by the IOBLPSO and by other methods [4, 21, 51], though it has moderate interaction effect. However, in terms of *P* value, the interaction (rs380390, rs1363688) is the most statistically significant one among all detected SNP-SNP interactions, which might be the reason of it being frequently detected. This fact implies that IOBLPSO is capable of capturing SNP-SNP interactions with statistically significant *P* values, though its fitness function is the mutual information. The SNP-SNP interaction (rs1329428, rs9328536) [52, 53], rs10507949 [4], and rs10512174 [51] also have been identified in AMD association studies, but their functions are still unclear. Other SNPs, that is, rs210758, rs223607, and rs718263, are the first time being identified. Further studies with the use of large-scale case-control samples are needed to confirm whether these SNPs have true associations with AMD. We hope that, from these results, some clues could be provided for the exploration of causative factors of AMD.

## 4. Conclusions

Detection of SNP-SNP interactions is believed to be important in understanding underlying mechanism of complex diseases. In this study, we proposed an improved opposition-based learning particle swarm optimization, or IOBLPSO, to detect SNP-SNP interactions. To the best of our knowledge, IOBLPSO is the first PSO based method to detect SNP-SNP interactions among possible SNP combinations. Highlights of IOBLPSO are the introduction of three strategies: OBL, dynamic inertia weight, and a postprocedure. Among them, OBL is the core, which is presented in the stage of updating particle experiences and common knowledge of swarm, not only for enhancing the global explorative ability, but also for avoiding premature convergence. Dynamic inertia weight is computed before the stage of updating particle velocities to allow particles to cover a wider search space while the considered SNP is likely to be a random SNP and to converge on promising regions of the search space while capturing a highly suspected SNP. The postprocedure is introduced as the final stage for carrying out a deep search in highly suspected SNP sets. Experiments of IOBLPSO are performed on lots of simulation data sets under the evaluation measures of detection power and computational complexity. Results demonstrate that IOBLPSO is promising for the detection of all simulation models of SNP-SNP interactions. IOBLPSO is also applied on a real AMD data set, results of which not only show the strength of IOBLPSO on real applications, but also capture important features of genetic architecture of AMD that have not been described previously. These features might provide new clues for biologists on the exploration of AMD associated genetic factors.

IOBLPSO might be an alternative to existing methods for detecting SNP-SNP interactions and has several merits. First, IOBLPSO is easy to be implemented, and its time costs can be estimated and controlled. Second, OBL and other two strategies help to improve the performance of IOBLPSO. Third, mutual information is effective in measuring SNP-SNP interactions. Fourth, compared with other methods, IOBLPSO needs less particles and/or iterations to obtain higher detection power, implying that IOBLPSO can handle large scale data sets for GWAS and its scalability is better than others. Though IOBLPSO is a beneficial exploration in the detection of SNP-SNP interactions, it still has several limitations; for example, multiple SNP-SNP interactions in a data set are not considered simultaneously; IOBLPSO is sensitive to those SNPs that display strong main effects. Furthermore, recent advancements in sequencing technology have enabled the sequencing of the whole-exome or even whole-genome of a cohort. The rare or de novo mutations resulting from these experiments should be considered. For example, Wu et al. recently proposed a bioinformatics method called SPRING for prioritizing candidate mutations [54]. It is therefore interesting to consider the problem of interactive effects of such de novo mutations. Limitations of IOBLPSO, as well as this new research hotspot, will inspire us to continue working in the future.

## Figures and Tables

**Table 1:** Average running time (seconds) of compared methods on simulation data sets. Experiments are conducted with Intel Xeon 2.00 GHz CPUs and 6 GB of RAM running Microsoft Windows XP Professional x64 Edition 2003 Service Pack 2 for computational complexity analysis.

Methods	BOOST	AntEpiSeeker	SNPRuler	TEAM	DBM-PSO	ISO	IOBLPSO
Running time	0.36	1146.60	1.56	13.14	68.73	13.40	20.75

**Table 2:** Detected SNP-SNP interactions associated with AMD. *P* values of detected SNP-SNP interactions before Bonferroni correction, as well as their linkage disequilibrium (LD) correlation coefficients *r* ^2^, are also recorded. The SNPs in different SNP-SNP interactions have low LD.

SNP	Gene	Chromosome	Times	Mutual information value		*P* value	LD (*r* ^2^)
				Individual	Interaction		
rs380390	*CFH *	1	1	**0.1412**	**0.2955**	5.0006*e* − 08	0.0089
rs1374431	*N/A *	2		0.0198			

rs380390	*CFH *	1	2	**0.1412**	0.2949	5.7995*e* − 08	0.0015
rs2402053	*N/A *	7		0.0476			

rs1329428	*CFH *	1	2	0.1218	0.2853	3.0012*e* − 08	0.0019
rs9328536	*MED27 *	9		0.0563			

rs380390	*CFH *	1	2	**0.1412**	0.2777	1.4359*e* − 07	0.0050
rs10512174	*ISCA1 *	9		0.0844			

rs380390	*CFH *	1	2	**0.1412**	0.2775	1.9451*e* − 07	0.0018
rs718263	*NCALD *	8		0.0471			

rs380390	*CFH *	1	1	**0.1412**	0.2760	1.9798*e* − 07	0.0019
rs223607	*N/A *	6		0.0079			

rs380390	*CFH *	1	**8**	**0.1412**	0.2752	1.1381**e** − 09	0.0031
rs1363688	*N/A *	5		0.0949			

rs380390	*CFH *	1	1	**0.1412**	0.2678	6.4999*e* − 07	0.0013
rs210758	*N/A *	4		0.0131			

rs380390	*CFH *	1	1	**0.1412**	0.2641	2.4273*e* − 07	0.0040
rs10507949	*N/A *	13		0.0948			
